# Machine learning on alignment features for parent-of-origin classification of simulated hybrid RNA-seq

**DOI:** 10.1186/s12859-024-05728-3

**Published:** 2024-03-12

**Authors:** Jason R. Miller, Donald A. Adjeroh

**Affiliations:** 1 Department of Computer Science, Mathematics, Engineering, Shepherd University, Shepherdstown, WV USA; 2 EVOGENE, Department of Biosciences, University of Oslo, Oslo, Norway; 3 Lane Department of Computer Science and Electrical Engineering, West Virginia University, Morgantown, WV USA

**Keywords:** Machine learning, RNA-seq, Allele-specific expression, Sequence alignment

## Abstract

**Background:**

Parent-of-origin allele-specific gene expression (ASE) can be detected in interspecies hybrids by virtue of RNA sequence variants between the parental haplotypes. ASE is detectable by differential expression analysis (DEA) applied to the counts of RNA-seq read pairs aligned to parental references, but aligners do not always choose the correct parental reference.

**Results:**

We used public data for species that are known to hybridize. We measured our ability to assign RNA-seq read pairs to their proper transcriptome or genome references. We tested software packages that assign each read pair to a reference position and found that they often favored the incorrect species reference. To address this problem, we introduce a post process that extracts alignment features and trains a random forest classifier to choose the better alignment. On each simulated hybrid dataset tested, our machine-learning post-processor achieved higher accuracy than the aligner by itself at choosing the correct parent-of-origin per RNA-seq read pair.

**Conclusions:**

For the parent-of-origin classification of RNA-seq, machine learning can improve the accuracy of alignment-based methods. This approach could be useful for enhancing ASE detection in interspecies hybrids, though RNA-seq from real hybrids may present challenges not captured by our simulations. We believe this is the first application of machine learning to this problem domain.

**Supplementary Information:**

The online version contains supplementary material available at 10.1186/s12859-024-05728-3.

## Background

RNA sequencing is a ubiquitous tool in molecular biology [1, 2]. The technology can detect gene transcription and quantify RNA abundance [2, 3]. Paired short reads, as generated by Illumina sequencing machines, constitute the vast majority of RNA-seq in public databases [1], and several software tools specialize in mapping short reads to references. Mappers such as Salmon [4] and Kallisto [5] use alignment-free algorithms, while mappers such as Bowtie2 [6, 7] and STAR [8, 9] are alignment-based. A comparison of these two approaches found that alignment-free methods are faster, and are equally accurate on simulated data, but fail to achieve the accuracy of alignment-based methods for quantification of human RNA-seq [10]. Various short-read aligners have been tested and compared for many applications [11–17]. Aligners such as HiSat2 [18, 19] and STAR [8, 9] specialize in mapping RNA-seq to genome sequences, a task that requires virtual splicing of read sequences into exons and generation of alignments that skip over introns in the reference.

Differential expression analysis (DEA) is the statistical and comparative analysis of RNA-seq quantities between environmental conditions, organisms, tissues, or other factors [3, 20, 21]. As a special case, DEA is used to detect imbalanced transcription of the maternal and paternal alleles in a diploid genome. The imbalance phenomenon is called allele-specific expression (ASE) [22]. Possible epigenetic mechanisms include genomic imprinting by DNA methylation or chromatin modification [23]. ASE has been associated with cancer and other human diseases [22]. ASE may be of evolutionary importance, as it is seen in interspecies hybrids of animals and plants [24]. ASE has been detected in the Neanderthal genes inherited by some modern humans [25]. ASE has been documented in seeds of plant hybrids, including hybrids of model organisms [26] as well as important crops [27]. MetaImprint [28], the Plant Imprinting Database [29], and ASMdb [30] are examples of databases of imprinted genes.

When using RNA-seq for ASE detection, a critical step is the computational association of each RNA sequence with its putative source allele. At least three basic approaches have been used to accomplish this. (1) A first approach aligns reads to a concatenation of two parental references, trusting the aligner to choose the better target. This was done in a study of crosses involving three species of the flowering plant *Arabidopsis*, *A. thaliana*, *A. lyrata*, and *A. halleri* [31]. In this case, references were available for only two of the three species, but a reference transcriptome for the third species was generated ad hoc by applying the Trinity [32] assembler to RNA-seq from that species. (2) A second approach aligns each read pair to each parental reference separately, then compares the quality of both alignments. This was used in two studies of crosses between ecotypes of *Arabidopsis thaliana* [26, 33]. In these studies, each parent’s transcriptome reference was computed by aligning its RNA-seq reads to a common reference, then customizing that reference with consensus polishing software [34]. (3) A third approach aligns reads to a single reference to bin them by gene, and then analyzes the reads for known single-nucleotide polymorphisms (SNPs) between the parental alleles of each gene. This approach has been used in many studies including studies of *Arabidopsis thaliana* [35, 36] and *Mus musculus* [37] intra-species crosses, and of the mule interspecies hybrid [38, 39]. This general approach has also been implemented in software [40, 41].

Machine learning has been used to generate pairwise sequence alignments [42, 43]. To our knowledge, it has not been used to post-process the output of standard sequence aligners.

We propose a machine learning approach to binning RNA-seq reads for the purpose of ASE detection in interspecies hybrid crosses. Our approach is inspired by trio binning, a technique that assigns DNA reads to either parental haplotype before computing a haplotype-resolved genome assembly of their cross [44]. We train a binary classifier to assign each RNA-seq read pair to its correct parent by analyzing features of its alignments to both parental references. Once trained, the classifier could be deployed on RNA-seq from the hybrid cross to assist ASE detection. Given the alignment of one read pair to both parental references, the classifier would choose the more likely parental alignment, which would indicate the most likely allele and gene of origin. Our results indicate that using a combination of an aligner plus machine learning is more accurate than using an aligner by itself. The resulting boost in parent-of-origin classification accuracy offers potential to boost the quality of ASE detection.

## Results

**Fig. 1:**
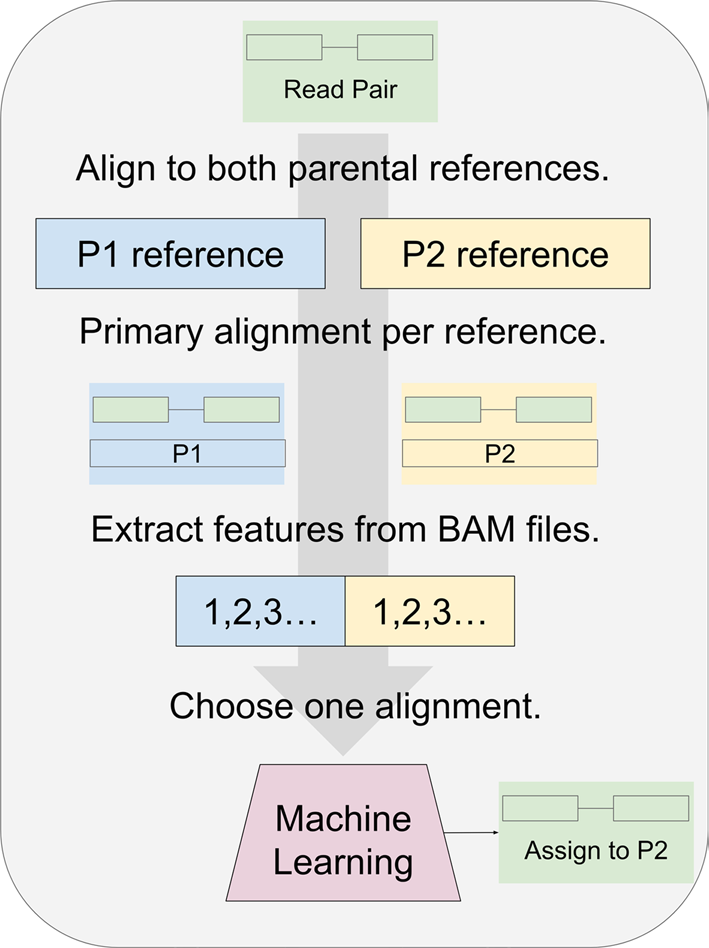
Process for classifying RNA-seq by machine learning. For training the model, read pairs are obtained from parents P1 and P2. Each read pair is aligned to the P1 and P2 references separately. A filter removes all but the primary alignment per read pair. The process can use transcriptomes or genomes as references, and any suitable aligner. For read pairs that align to both P1 and P2, features are extracted and given to the machine learning model. Initially, the model is given the true parent label per read pair and trained to predict this. After training, read pairs from the hybrid cross are given instead. In this illustration, after a hybrid read pair (green) is aligned to the P1 (blue) and P2 (yellow) references, the trained model (red) chooses P2 as the more likely source. For most of this study, the model was a random forest binary classifier. Read pairs classified by this process could be binned by gene and quantified to detect allele-specific gene expression in the hybrid

We developed the process illustrated in Fig. 1. The training pipeline uses RNA-seq read pairs from both parents, as well as a reference genome or transcriptome from each parent. Every read pair is aligned to both parental references. Features are extracted from the alignments by a Python script that parses the aligner output. A machine-learning model is trained to predict the parent-of-origin per read pair based on the alignment features. The prediction pipeline applies a similar process to read pairs from the hybrid offspring. In this case, the unknown parent-of-origin per read pair must be predicted. To assess model accuracy in hybrids, we simulated hybrid data by combining real RNA-seq data from both parents in equal quantities. Possible limitations of the simulation are addressed in Discussion.

We searched INSDC databases [45] for any two organisms each having (1) a reference genome assembly, and (2) a reference transcriptome assembly, and (3) at least one RNA-seq dataset. We filtered for organisms that are known to hybridize and belong to the same genus. We filtered further for organisms having RNA-seq datasets that were comparable by read length and sample collection methods. In some cases, after low map rates were seen, different RNA-seq data was sought out. The search yielded data for: (1) two species of the flowering plant genus *Arabidopsis*; (2) two species of the cultivated plant genus *Brassica*; (3) two strains of the mouse species *Mus musculus*; and (4) two species of the equine genus *Equus.*

We selected mapping software packages based on the software’s ability to generate SAM/BAM files [46] that include the required and optional fields required for our alignment feature extraction process. For mapping to reference transcripts, we selected the aligners Bowtie2 [6, 7] and STAR [8, 9], and configured STAR for mapping to RNA references lacking introns. For mapping to reference genomes, we selected HiSat2 [18, 19], which incorporates Bowtie2, and STAR using STAR’s default configuration for mapping to DNA containing introns. Results from the bwa mem [47] aligner and the Salmon [4] mapper were summarized but not used for machine learning because their outputs did not fully satisfy the requirements of our feature extraction process.

We filtered alignments to require the reads map as a proper pair, to retain only the primary alignment per pair, and to have non-zero map quality score, i.e. mapq >  = 1. More stringent mapq filters would have eliminated large quantities of multiply mapped reads, which are integral to our process. Analysis of alignments to transcriptomes showed that a mapq >  = 40 filter eliminates many reads having an alignment that achieved the maximum alignment score (Additional file 2: Table S14) and all alignments to many transcripts (Additional file 2: Table S15).

For each genus, RNA-seq read pairs from two parental organisms were combined in equal quantities. The 50:50 ratio was chosen based on our assumption that parental expression in real hybrid data is about even for most genes, and to discourage models from incorporating priors i.e. favoring predictions of the majority class. The 50:50 combination represents a simple model of real hybrid RNA-seq lacking any ASE, overlooking complicating factors such as differential isoform abundances between parental alleles.

Read pairs were mapped to both parental references, using either both transcriptomes or both genomes as references. For machine-learning approaches, fifty-three features were selected to represent the pairwise sequence alignment generated by one aligner of one RNA-seq read pair to two parental references, where both references were either transcriptomes or genomes. The features are listed in Table 1. A portion of the read pair were used to train a random forest model [48]. A separate portion of the read pairs were used to quantify the predictive performance of the trained model.

**Table 1:** Alignment features used for machine learning

Feature type	Extraction	Technical notes
**(A) Per read alignment**		
AS: Alignment Score ED: Edit Distance MM: Mismatch count HQMM: HQ mismatch count GO: Gap Open count GE: Gap Extend count INS: Insertion count HQINS: HQ insertion count DELS: Deletion count HQDEL: HQ deletion count	Taken from: ● P1 R1, ● P1 R2, ● P2 R1, ● P2 R2 10 feature types, 40 features total	High-quality (HQ) means that the base call quality score is the maximal value. The HQ requirement was applied to the one base involved in a mismatch or insertion, and to the two surrounding bases for deletion. INS or DEL refer to an extra or missing base in the read, respectively. GO is the number of separate indels, and GE is the number of bases in indels
**(B) Compare totals per parent**		
AS diff ED diff MM diff HQMM diff GO diff GE diff INS diff DELS diff HQINS diff HQDEL diff MAT diff	Subtract (P1 R1 + P1 R2) from (P2 R1 + P2 R2)	Each difference represents the sum over the read pair alignments to parent 2 minus the equivalent sum for parent 1 MAT is the matched base count. See **A)** for other feature types
**(C) Compare spans per parent**		
Span diff	Subtract P1 span from P2 span	Span is the length of the read pair alignment along the reference
**(D) The better alignment score**		
Parent choice	Compare P1 to P2	Use -1 or + 1 to indicate whether P1 or P2 had the greater alignment score, respectively, or 0 if tied

After an aligner, such as Bowtie2, aligned an RNA-seq read pair to both parental references, the primary alignment per reference was selected based on flags in the aligner output. Then, 53 features were extracted for machine learning. **A)** These 10 features, taken directly from the aligner output, describe the alignment of one read to one reference. Four sets of 10 features were extracted to represent reads R1 and R2 aligned to references of parents P1 and P2. **B)** These features combine and compare the features in part A. **C)** This compares the lengths of the read pair’s projection onto each parental reference. **D)** This compares the overall alignment scores, generated by the aligner, of the read pair to each parental reference.

For comparisons, mappers were also used without machine learning to predict parent-of-origin. One comparison ran each mapper against both parental references together and extracted parent-of-origin from the mapper’s primary alignment choice. A second comparison ran each mapper against both parental references separately and decided parent-of-origin (or a tie) from the higher alignment score. A third possibility, relying only on reads that aligned to one parent only, was found to be ineffective (Additional file 2: Table S13) and was not used.

### Results with genus *Arabidopsis*

This experiment used public data from *A. lyrata* and *A. halleri*, two species of the flowering plant genus *Arabidopsis*. The transcriptome results are characterized in Table 2. As shown in column A, we achieved 73% accuracy by running Bowtie2 on the concatenation of parental references. As shown in column B, we achieved 81% accuracy by running Bowtie2 twice, once on each parent reference, then choosing the one alignment having the higher alignment score. As shown in column C, we achieved 95% accuracy by applying the random forest post-process to the alignment generated for column B. Out of these three approaches, the random forest achieved the highest performance by all measures including accuracy, F1-score, and MCC. Columns D, E, and F show the same experiment repeated using the STAR aligner configured for transcript alignments. Again, the highest performance was achieved with random forest. Column G characterizes the performance of the alignment-free software called Salmon, and column H characterizes the bwa mem software, whose output lacked a feature required for our machine learning. The model performance in this table and subsequent tables was measured on a set of read pair alignments reserved for testing and withheld from training. Five-fold cross-validation results on the training sets predicted similar results with low variance; see supplement S2-B.

**Table 2:** Classification Performance with Reference Transcripts of genus *Arabidopsis*

*Arabidopsis* RNA	A Bowtie2	B Bo_AS	C Bo_RF	D STAR	E St_AS	F St_RF	G Salmon	H bwa
Accuracy	72.7%	81.0%	95.0%	73.0%	80.9%	88.5%	70.8%	75.2%
Sensitivity	56.9%	70.7%	90.6%	56.0%	69.9%	87.2%	48.1%	60.9%
Specificity	88.5%	91.3%	99.5%	90.0%	91.9%	89.9%	93.5%	89.0%
Precision	83.2%	89.1%	99.4%	84.9%	89.6%	89.6%	88.0%	84.7%
F1-score	67.5%	78.8%	94.8%	67.5%	78.5%	88.3%	62.2%	70.9%
MCC	0.478	0.634	0.904	0.489	0.633	0.770	0.466	0.521
AUPRC	–	–	99.5%	–	–	96.5%	–	–
AUROC	–	–	99.4%	–	–	96.2%	–	–
Pos Pref	34.2%	39.7%	45.6%	33.0%	39.0%	48.6%	27.3%	36.0%
Ties	–	14.0%	–	–	14.7%	–	–	–

Performance metrics for parent-of-origin classification in *Arabidopsis*. In all seven approaches, RNA-seq read pairs were assigned to either of two reference transcriptomes. Whether used with Bowtie2 or STAR, the random forest method demonstrated superior performance. For the sake of directional statistics like sensitivity, species *A. lyrata* and *A. halleri* were designated as the negative and positive classes, respectively. (A) Parent chosen by the Bowtie2 aligner. (B) Parent chosen by comparing Bowtie2 alignment scores. (C) Parent chosen by the random forest classifier using Bowtie2 alignment features. (D, E, F) Similar to columns A, B, C, but using the STAR aligner, configured to avoid splicing. (G, H) Parent chosen by Salmon or bwa, respectively

To evaluate alternate machine learning architectures, the Bowtie2 experiment was repeated with three other architectures: a gradient boosting classifier, a support vector machine, and a multi-layer perceptron. The results (Additional file 1: Table S1) did not exceed those of Table 2. To evaluate whether more trees would help the random forest, the experiment was repeated after increasing the number of trees within the random forest. The results (Additional file 1: Table S2) did not exceed those of Table 2. Therefore, the default random forest model was used for the remaining experiments.

Whereas Table 2 showed results with the transcriptome references, Table 3 shows the results using the genome references and splice-aware alignments. The highest performance, as measured by accuracy, F1, or MCC, was achieved with the machine-learning post-process (columns C and F). The overall highest accuracy on *Arabidopsis* data was achieved with HiSat2 plus random forest, and in this case, the accuracy climbed from 73% with the aligner alone to 95% with machine learning.

**Table 3:** Classification Performance with Reference Genomes of genus *Arabidopsis*

*Arabidopsis* DNA	A HiSat2	B Hi_AS	C Hi_RF	D STAR	E St_AS	F St_RF	G bwa
Accuracy	73.3%	82.8%	94.5%	72.9%	81.4%	88.6%	76.4%
Sensitivity	50.1%	72.1%	91.2%	49.6%	69.9%	87.6%	56.1%
Specificity	96.4%	93.5%	98.3%	96.2%	93.0%	89.7%	96.7%
Precision	93.4%	91.7%	98.1%	92.9%	90.9%	89.5%	94.4%
F1-score	65.2%	80.7%	94.5%	64.6%	79.0%	88.5%	70.4%
MCC	0.525	0.671	0.897	0.518	0.646	0.773	0.578
AUPRC	–	–	99.3%	–	–	96.5%	–
AUROC	–	–	99.2%	–	–	96.2%	–
Pos Pref	26.8%	39.3%	46.5%	26.7%	38.4%	48.9%	29.7%
Ties	–	13.3%	–	–	14.7%	–	–

Performance metrics for parent-of-origin classification in *Arabidopsis*. In all six approaches, RNA-seq read pairs were assigned to either of two reference genomes. Whether used with HiSat2 or STAR, the random forest led to superior accuracy, F1, and MCC. For the sake of directional statistics like sensitivity, species *A. lyrata* and *A. halleri* were designated as the negative and positive classes, respectively. (A) Parent chosen by the HiSat2 aligner. (B) Parent chosen by comparing HiSat2 alignment scores. (C) Parent chosen by the random forest classifier using HiSat2 alignment features. (D, E, F) Similar to columns A, B, and C but using the STAR aligner, configured for splicing. (G) Parent chosen by bwa

Mild class imbalance is seen in Table 2. For example, in column C, sensitivity < specificity and precision > recall. (Recall and sensitivity are the same in binary classification). Also, the positive-preference statistics show less than 50% of read pairs assigned to the positive class, though the sample contained 50% from each class. These statistics reveal a bias for choosing the negative class (*A. lyrata*) and a tendency to mistakenly assign *A. halleri* read pairs to the *A. lyrata* parent. Investigation showed the mistakes were non-random, with a few transcripts attracting large portions of the mistaken alignments. Class bias is seen again in Table 3. In both tables, the deviation from 50% was reduced in the machine learning predictions, compared to that of the aligners. Possible causes and mitigations for the bias will be addressed in the “Discussion”.

To test whether the trained models could generalize to parts of the references it had not seen, a model was trained and evaluated on different parts of the references. This experiment used HiSat2 alignments and Arabidopsis genomes. Alignments to chromosomes 6, 7, and 8, comprising approximately 20% of alignments, were withheld from training and used only for testing. The resulting performance statistics (Additional file 2: Table S9) were comparable to those in Table 3, column C. This result indicates that the models were not overfitting particular alignments.

### Results with genus *Brassica*

This experiment used public data from *B. oleracea* and *B. rapa*, two species in genus *Brassica* which includes many cabbage-like cultivars consumed by humans. The transcriptome and genome results are characterized in Table 4 and 5 respectively. Compared to the aligners, the trained models showed comparable or better accuracy, F1, and MCC values.

**Table 4:** Classification Performance with Reference Transcripts of genus *Brassica*

*Brassica* RNA	A Bowtie2	B Bo_AS	C Bo_RF	D STAR	E St_AS	F St_RF	G Salmon	H bwa
Accuracy	89.3%	92.1%	93.9%	91.8%	92.2%	93.8%	89.4%	89.6%
Sensitivity	92.8%	94.7%	92.8%	94.3%	94.6%	93.7%	89.3%	92.9%
Specificity	85.9%	89.5%	94.9%	89.2%	89.8%	93.9%	89.6%	86.3%
Precision	86.8%	90.1%	94.8%	89.7%	90.3%	93.9%	89.6%	87.1%
F1-score	89.7%	92.3%	93.8%	92.0%	92.4%	93.8%	89.4%	89.9%
MCC	0.789	0.844	0.877	0.836	0.846	0.876	0.788	0.793
AUPRC	–	–	–	–	–	98.4%	–	–
AUROC	–	–	98.4%	–	–	98.4%	–	–
Pos Pref	53.4%	52.6%	49.0%	52.6%	52.4%	49.9%	49.8%	53.3%
Ties	–	3.3%	–	–	5.0%	–	–	–

Performance metrics for transcript-based parent-of-origin classification in *Brassica*. Whether used with Bowtie2 or STAR, the random forest improved the accuracy, F1, and MCC. For directional statistics, species *B. rapa* and *B. oleracea* were considered the negative and positive classes, respectively. (A, B, C) Using the Bowtie2 aligner, a parent was chosen by the aligner, or by comparing alignment scores, or by the random forest, respectively. (D, E, F) Similar to A, B, and C but using the STAR aligner, configured to avoid splicing. (G, H) Parent chosen by Salmon or bwa, respectively

**Table 5:** Classification Performance with Reference Genomes of genus *Brassica*

*Brassica* DNA	A HiSat2	B Hi_AS	C Hi_RF	D STAR	E St_AS	F St_RF	G bwa
Accuracy	93.0%	93.1%	95.1%	94.5%	92.7%	94.3%	94.8%
Sensitivity	95.8%	95.7%	94.7%	97.1%	94.8%	94.0%	97.3%
Specificity	90.3%	90.6%	95.6%	91.9%	90.5%	94.6%	92.3%
Precision	90.8%	91.0%	95.6%	92.3%	90.9%	94.6%	92.6%
F1-score	93.2%	93.3%	95.1%	94.6%	92.8%	94.6%	94.9%
MCC	0.862	0.863	0.903	0.890	0.854	0.886	0.897
AUPRC	–	–	99.0%	–	–	98.5%	–
AUROC	–	–	98.9%	–	–	98.5%	–
Pos Pref	52.8%	52.6%	49.5%	52.6%	52.2%	49.7%	52.5%
Ties	–	3.4%	–	–	4.8%	–	–

Performance metrics for genome-based parent-of-origin classification in *Brassica*. Whether used with HiSat2 or STAR, the random forest improved the accuracy, F1, and MCC. For directional statistics, species *B. rapa* and *B. oleracea* were considered the negative and positive classes, respectively. (A, B, C) Parent chosen by the HiSat2 aligner, or by comparing HiSat2 alignment scores, or by the random forest using HiSat2 alignment features, respectively. (D, E, F) Similar to columns A, B, and C but using the STAR aligner, configured for splicing. (G) Parent chosen by bwa

The model did not boost the results of STAR on *Brassica* DNA. The overall highest accuracy with *Brassica* was achieved with HiSat2 plus random forest, and in this case, the accuracy rose from 93% with the aligner alone to 95% with machine learning. Each mapping software package showed better performance with the *Brassica* data (Tables 4 and 5), than with the *Arabidopsis* data (Tables 2 and 3) and the gains by machine learning were smaller. A possible factor is the smaller numbers of alignment score ties in *Brassica* (3%-5%) than in *Arabidopsis* (13%-15%). It may be that the random forest has the most effect when the aligners generate many equally good (or equally bad) alignments between the two parental references.

Mild class imbalance is seen in the *Brassica* results, though the positive preference was closer to 50% than in *Arabidopsis*. The alignment-based methods (columns A, B, D, and E) showed the most imbalance between sensitivity and specificity, and these imbalances were reduced by the machine-learning post-process (columns C and F).

### Results with genus *Mus*

This experiment used public data from *Mus musculus*, the mouse species that serves as a model organism for mammalian genetics. The B6 and D2 strains are inbred laboratory strains of the same species. Parent-of-origin classification accuracy was low, barely exceeding the 50% expected by random guessing. Accuracy did not exceed 57% by any method tested. These two strains harbor five to ten fold less sequence divergence than the other genera tested here, as shown by the mash [49] similarity scores (Additional file 2: Table S10). These results suggest that within-species hybrids do not present enough genomic variation for parent-of-origin classification. For this reason, the *Mus* results are shown in supplement (Additional file 1: Tables S3 and S4), and the procedure is not recommended for crosses between very similar genotypes. Nevertheless, even on this dataset, the random forest post-processor added value, achieving 56%-57% accuracy, compared to 53%-56% achieved by the aligners directly. The random forest predictions showed positive class bias (toward D2) using transcript references, but negative class bias (toward B6) using genome references.

### Results with genus *Equus*

This experiment used public data from the genus *Equus*. The equine species of horse, *E. caballus,* and donkey, *E. asinus,* can hybridize to yield a mule or hinny, depending on which parent is male or female [50]. Results are shown in Tables 6 and 7. The bwa aligner performed best using the *Equus* genomes; it remains for future work to discover whether our machine learning process could be adapted to post-process bwa output, which does not include all the currently required features. The machine-learning method provided the best performance using transcriptomes, and second and third best using genomes. These results indicate that our method is suitable for interspecies hybrids of animals as well as plants.

**Table 6:** Classification Performance with Reference Transcripts of genus *Equus*

Equus RNA	A Bowtie2	B Bo_AS	C Bo_RF	D STAR	E St_AS	F St_RF	G Salmon	H bwa
Accuracy Sensitivity Specificity Precision F1-score MCC AUPRC AUROC Pos Pref Ties	73.0% 78.4% 67.6% 70.8% 74.4% 0.463 – – 55.4% –	76.7% 75.2% 78.3% 77.6% 76.4% 0.535 – – 48.4% 38.6%	81.3% 91.1% 71.6% 76.2% 83.0% 0.637 92.0% 91.4% 59.7% –	78.8% 78.4% 79.1% 79.1% 78.7% 0.576 – – 49.6% –	77.2% 75.4% 78.9% 78.2% 76.8% 0.544 – – 48.2% 38.6%	85.8% 80.1% 91.5% 90.4% 84.9% 0.720 93.9% 93.8% 44.3% –	69.1% 54.8% 83.4% 76.8% 63.9% 0.399 – – 35.7% –	68.6% 76.7% 60.4% 66.0% 70.9% 0.376 – – 58.2% –

By many measures including accuracy, F1, and MCC, the random forest performance surpassed that of the other methods tested. For directional statistics, donkey and horse were considered the negative and positive classes, respectively. (A, B, C) Using the Bowtie2 aligner, a parent was chosen by the aligner, or by comparing alignment scores, or by the random forest, respectively. (D, E, F) Similar to A, B, and C but using the STAR aligner, configured to avoid splicing. (G, H) Parent chosen by Salmon or bwa, respectively

**Table 7:** Classification Performance with Reference Genomes of genus *Equus*

Equus DNA	A HiSat2	B Hi_AS	C Hi_RF	D STAR	E St_AS	F St_RF	G bwa
Accuracy Sensitivity Specificity Precision F1-score MCC AUPRC AUROC Pos Pref Ties	79.1% 78.4% 79.9% 79.6% 79.0% 0.582 – – 49.3% –	78.9% 78.2% 79.4% 79.2% 78.7% 0.576 – – 49.4% 33.8%	84.0% 90.6% 77.4% 80.0% 85.0% 0.686 93.7% 93.5% 56.6% –	79.2% 78.6% 79.9% 79.6% 79.1% 0.584 – – 49.4% –	78.2% 77.5% 79.0% 78.6% 78.0% 0.564 – – 49.3% 37.0%	86.0% 81.9% 90.1% 89.2% 85.4% 0.723 94.0% 93.9% 45.9% –	88.9% 91.5% 86.3% 87.0% 89.2% 0.780 – – 52.9% –

By many measures including accuracy, F1, and MCC, the random forest performance surpassed that of the other methods tested. For directional statistics, donkey and horse were considered the negative and positive classes, respectively. (**A, B, C)** Parent chosen by the HiSat2 aligner, or by comparing HiSat2 alignment scores, or by the random forest using HiSat2 alignment features, respectively. (**D, E, F)** Similar to columns A, B, and C but using the STAR aligner, configured for splicing. (**G)** Parent chosen by bwa

The random forest post-process introduced mild levels of class bias on the *Equus* data, though the direction varied. Positive preference increased when the random forest was used with Bowtie2 or HiSat2 but decreased when used with STAR. This indicates that, at least on these data, the bias is a computational artifact.

### Results summary and generalization

**Fig. 2:**
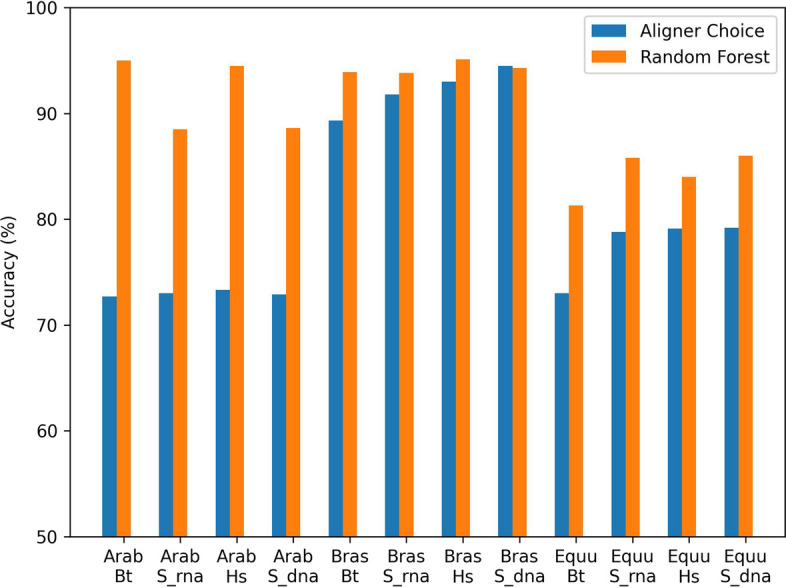
Summary of accuracy gains. Bars show the parent-of-origin prediction accuracy on simulated hybrid reads. Blue: Read pair origin predicted by aligner’s choice of one best alignment to the concatenation of parental references. Orange: Origin predicted by a model trained on one portion of those alignments and tested on another portion. In the X-axis labels, Arab = *Arabidopsis*, Bras = *Brassica*, and Equu = *Equus*, while Bt = Bowtie2, Hs = HiSat2, and S = STAR. Bowtie2 was used on RNA references, HiSat2 on DNA references, and STAR on both. The vertical axis ranges from 50% (guessing) to 100% (perfect)

The results on the three interspecies datasets are compared in Fig. 2. For each of the *Arabidopsis*, *Brassica*, and *Equus* datasets, the approach that achieved the highest accuracy is compared to the other approaches tested with the same aligner. On each dataset, the random forest accuracy surpassed that of relying on the alignment scores or the aligner by itself. On *Arabidopsis* and *Brassica*, the maximum accuracy was achieved using HiSat2 alignments to the genome reference, plus the random forest. On *Equus*, the maximum machine learning accuracy was achieved using STAR alignments to the genome reference, plus the random forest. Since no one approach worked best on all three datasets, it may be advantageous to experiment with several aligners, as we have done here, when using other datasets.

**Fig. 3:**
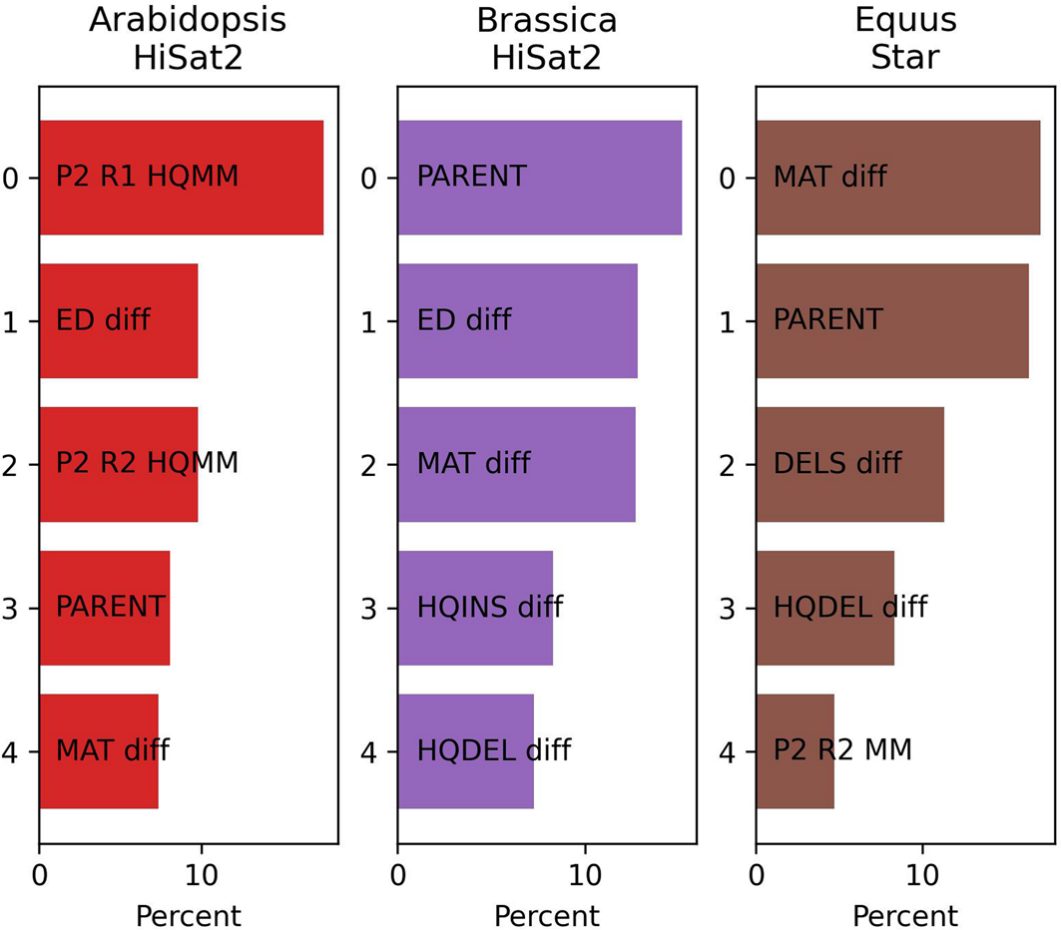
Alignment Features Ranked by Importance. Top five alignment features, ranked by importance, used by the random forest model. The three columns here correspond to those in Fig. 2. The figure indicates that different models relied on different features. Labels like “P2 R1” refer to the alignment of read 1 to parent 2. Labels with “HQ” count only those events that involve a maximal base call quality score in the read. Labels with “diff” include the difference between parent 2 and 1 alignments. Labels with “MM” and “MAT” refer to mismatched or matched base counts, respectively. Labels with “INS” or “DEL” refer to bases inserted or deleted in the read, respectively. The label “PARENT” indicates a feature that was 1 if P2 had the better combined alignment score, or -1 if P1 had the better score, or 0 if they were tied

Unlike many machine learning models, random forest models can be interrogated for indications of which features were most important. Figure 3 illustrates the top five features reported for the three most performant models in this study. See also Additional file 1: Table S5. The different rankings in this comparison indicate that the feature importance varied with the dataset and the aligner. However, the features in the intersection of these three lists are Parent (a number indicating the parent with higher alignment score) and MAT diff (the P2-P1 difference in matched bases). Also, there was at least one HQ feature in each list. Our HQ features count problems (mismatches, insertions, deletions) that involve positions in reads assigned the maximal quality score by the sequencing instrument software.

In all the experiments shown so far, each model was trained on one set of read pairs and tested on another. However, each test set was carved from the same RNA-seq runs as its cognate training set. In an experiment on actual hybrids, the training RNA-seq from the parents would come from different sequencing libraries and sequencing runs compared to the test RNA-seq from the hybrid. To determine whether our models would generalize, we next tested on RNA-seq runs other than those used for training.

We tested the *Equus* RNA model. Two additional parental RNA-seq runs from the same *Equus* project but for different individuals, i.e.*,* a different horse and a different donkey, were aligned to the parental transcriptomes with Bowtie2. The random forest model that was trained on the primary runs was used without retraining to make predictions on the secondary runs. The model achieved 82.5% accuracy, 0.659 MCC, and 91.4% AUROC on the secondary runs (Additional file 1: Table S6). Overall, the model performed similarly whether evaluated with the primary or secondary runs. Thus, the model was able to generalize and maintain accuracy with RNA-seq runs other than those on which it was trained.

### Results: case study

We sought to demonstrate how this computational process could be applied for biology. A literature survey was conducted to find a study that had generated public RNA-seq datasets consisting of paired-end RNA-seq reads of 100 bases or more, derived from two parents plus their hybrid offspring, leading to a list of genes found to be in ASE. The closest match found was a study of pluripotent stem cells [51], that generated paired-end, 2 × 150-base RNA-seq for two horses, two donkeys, and two mules. Unfortunately for our application, the animals were not necessarily related, and the RNA source was unusual: adult fibroblast cells taken from ear and grown in culture. Also, ASE in these cells was not investigated and ASE is not known to occur in these cells. Despite the inability to verify any ASE predictions with this resource, three sequencing runs were downloaded, yielding 23.3 to 23.8 million read pairs per animal after trimming from each of one horse, one donkey, and one mule.

To establish an expectation for accuracy, read pairs from horse and donkey were aligned to a concatenation of the horse and donkey transcriptomes, with Bowtie2 choosing one best target per read pair. These read-to-parent assignments were 73% accurate with a 56% preference for the horse reference (Additional file 2: Table S12).

Next, the horse, donkey, and mule read pairs were aligned to the horse and donkey transcriptomes separately, with Bowtie2 choosing one best horse target, and one best donkey target, per pair. Map rates were consistently high: 91.1% of horse pairs, 89.2% of donkey pairs, and 89.8% of mule pairs (Additional file 2: Table S12). About 14% of read pairs mapped to one reference exclusively, but these maps favored the horse reference by 78% and were poor indicators of the true parent. Of read pairs that mapped to both references, the better alignment score indicated the true parent with 78% accuracy, incorporating random tie breaking for 35% of pairs.

Of read pairs that Bowtie2 had mapped to both horse and donkey separately, 1 million horse pairs and 1 million donkey pairs were split 80:20 into train and test sets. The model trained on the training set had 80% accuracy on the test set, with a 65% horse preference. These results indicated that model accuracy (80%) would exceed aligner accuracy (73%) and alignment score accuracy (78%) when applied to the mule read pairs. However, the model’s horse preference (65%) would have to be incorporated as the baseline.

Next, mule read pairs were mapped to the concatenated references using Bowtie2. The accuracy of parent assignment could not be determined, but the horse preference was observed to be 57%. Since Bowtie2 showed 56% horse preference on the horse and donkey reads, this result is consistent with a hypothesis of 49:51 donkey:horse allele origins of mule read pairs.

Finally, the model trained on horse and donkey reads was used to predict one parental allele for each mule read pair having an alignment to each parental reference. The accuracy of these predictions could not be determined, but they showed 66% horse preference. Since the model showed 65% horse preference on horse and donkey reads, this result is consistent with the above-stated hypothesis of 49:51 donkey:horse allele origins of mule read pairs. In summary, though model accuracy on the mule reads remains unknown, the model accuracy exceeded aligner accuracy on horse and donkey reads, and the level of allelic imbalance was similar between the model’s and the aligner’s predictions.

ASE detection relies on statistics and thresholds applied to mapped read counts, whether applied at the level of individual transcripts, individual genes, or entire transcriptomes. Our method boosted parent-of-origin accuracy on simulated hybrid data. Although we have yet to prove it on real hybrid data, our method has the potential to increase the sensitivity of ASE detection by increasing the number of correctly assigned read pairs from hybrids.

## Discussion

We demonstrated process improvement for one part of pipelines that study allele-specific expression (ASE) in hybrids. We examined several procedures for classifying paired short-read RNA-seq data according to their parent of origin. In most cases, the best performance was achieved by a novel process that applies machine-learning to features extracted from pairwise sequence alignments. We believe that this is the first application of machine learning to the problem of binning RNA-seq reads by parent-of-origin.

Using public data representing seven species in four genera, we trained classifiers to bin RNA-seq read pairs by their parent of origin. The reason for training on parental reads was that their true parent of origin was known in all cases. For evaluation, we used combinations of parental reads, again because their true parent of origin was known. Thus, the evaluations were conducted on simulated hybrid datasets composed of mixtures of real RNA-seq data.

We tested with one transcript aligner, one genome aligner, and one aligner that worked with transcripts or genomes. For each aligner, we evaluated three configurations: relying on the aligner to choose the parent of origin, or by choosing a parent based on the better alignment score, or by feeding alignment features into a machine-learning post-processor. In all our experiments, the post-processor boosted the performance of the aligner.

For machine learning, we used random forest models and interrogated them for feature importance. The top five features per model included one based on alignment scores and two that are independent of alignment scores. It appears that the models learned to rely on complementary features. The features that we extracted from alignments included imperfection counts (mismatches, indels), differences in imperfection counts, and the difference in match counts. The models were not given the actual match counts, read lengths, or alignment spans, because these might allow models to distinguish reads by their RNA-seq library or sequencing run. If models had focused on library-specific or run-specific features, they would not generalize to other RNA-seq data. We saw confirmation that our models could generalize when we trained and tested a model on one *Equus* RNA-seq dataset and then tested the trained model on another RNA-seq dataset. The model achieved similar performance on the second dataset.

We relied on simulations by combining real RNA-seq from two potential parents of a hybrid. Here we speculate on ways our simulated data might differ from real hybrid RNA-seq. First, real hybrid individuals inherit only one allele per gene per parent, but our simulated hybrid data represented up to four parental alleles per gene. Second, a real hybrid individual might express an allele or isoform not expressed in either parent. Third, RNA-seq from real hybrids may reflect novel genes generated, for example, by a mid-gene crossing-over event during meiosis in either parent’s germ cell. Any of these cases could have escaped our notice in our case study with real hybrid RNA-seq.

The fact that machine learning could improve on aligner accuracy should not be taken as criticism of aligners, which are general-purpose tools that have enabled many biological discoveries. We employed aligners for one specific task, parent-of-origin assignment, and used millions of training samples from each parent. We explored the use of machine learning as a post-process. It may be possible to tune or parameterize aligners for the specific task, as has been explored for other tasks [52, 53]. We did not find that any one aligner was best for all situations. Our experiments suggest that ASE investigators should test several aligners, then possibly select one whose alignments yield the highest accuracy among trained models.

In ASE studies, accurate quantification is key. Meta-analyses of published lists of ASE genes in *Arabidopsis* have noted little overlap between the lists [33, 54]. Other meta-analyses have called into question published claims of weak imprinting in humans and mice [55]. Simulations have shown that ASE discoveries are sensitive to underlying map bias [56] and read trimming [57]. Our method appears to increase the portions of RNA-seq reads assigned to the correct parent of a hybrid organism. This improvement could lead to improved sensitivity and specificity and thus higher confidence in ASE detection.

Map bias was seen in all our experiments. Map bias may result from different levels of completeness or quality between the two references, and from different degrees of sequence similarity between the references and the sequenced individuals. Algorithmic factors may also contribute, as indicated by differences between our results using different aligners on the same data. Regardless of its cause, map bias can induce false conclusions about ASE [56]. Ideally, our models would learn to overcome any bias in the aligner outputs. In fact, our models often reduced the bias but sometimes exacerbated it. We demonstrated how to measure the bias and incorporate it into ASE detection. In our mule experiment, where the bias was the most extreme, we simulated parental reads in 50:50 proportion but observed parental assignments in 35:65 proportion. Therefore, we used 35:65 as the baseline for ASE detection. Observing mule results close to this baseline, we accepted the null hypothesis of no ASE. Our mule analysis was performed at the whole transcriptome level, but it could be repeated at the level of individual genes or transcripts, and it could be enhanced by employing biological replicates and statistics.

It may be possible to reduce model bias by incorporating prior class weights to a model’s loss function. One such heuristic has been described by King and Zeng [58] and incorporated as an option in the fit function of scikit-learn models.

To put our approach into practice on a real hybrid organism, an experimenter would need to sequence RNA from one or more hybrids plus both of its parents, then align all the RNA-seq data to both parental references. Either two genome references or two transcriptome references could be used. (For organisms lacking trusted references, a reference transcriptome could be generated by de novo assembly of either parental RNA-seq dataset using e.g*.*, Trinity [32], with the limitation that this ad hoc reference would only reflect genes expressed by the parent.) The experimenter could choose one aligner from several by comparing their parent-of-origin accuracy on parental read pairs, as we have done. The experimenter would train and test a classifier on the parental RNA-seq alignments, then apply the trained classifier to the hybrid RNA-seq alignments, as in our mule case study. The experimenter would infer the allele of origin per hybrid RNA-seq read pair using its alignment to the parental reference predicted by the classifier. The counts per allele per gene could be given to any differential expression analysis pipeline for ASE detection. Implementation of such a pipeline is left for future work.

Our approach assigns the parent-of-origin and gene in one step. It seems common practice to assign the gene first, based on alignments, and the parental allele second, based on sequence variants. Comparisons are left for future work, but our approach of deciding the parent first allows for the creation of labeled training sets and the injection of machine learning.

## Conclusion

Random forest models were trained on alignment features extracted from RNA-seq paired-end reads aligned to reference transcriptomes or genomes. For each aligner tested, the model provided higher accuracy at parent-of-origin classification than the aligner by itself. This study establishes that machine learning can play a role in RNA-seq analysis of allele-specific gene expression in hybrids.

## Methods

All mapping software ran under Linux (Rocky 9.1) on the Saga computing cluster in Norway. Alignment files in SAM/BAM format were manipulated with samtools [46] version 1.16.1. Software compilations used GCC 11.3.0. The machine learning software ran inside Jupyter notebooks on Google CoLab Pro virtual computers with one CPU and 12 GB RAM.

Reference genome files were downloaded in FASTA format from Ensembl [59]. Ensembl provides a “primary_assembly” file when chromosome sequences are available. All the assemblies used here, except mouse b6, lacked chromosome assignments. For consistency, the “toplevel” files of scaffolds were used in all cases. Reference transcript files were downloaded from the cDNA directories corresponding to these genomes. The cDNA files contain intron-free transcript references. RNA-seq files were downloaded from NCBI SRA [60]. In every case, the SRA normalized file was selected to obtain original base call quality values. SRA files were processed with ‘fastq-dump –split-3’ from the SRA-Toolkit version 3.0.3 to create FASTQ files. Where two database accessions are given below, the first is a general one given in the publication and the second indicates the data subset used here. See also Additional file 2: Table S7.

1. *Arabidopsis*. We used the *A. halleri* reference with accession GCA_900078215 (no publication) and the *A. lyrata* reference with accession GCA_000004255.1 [61]. (Newer references for *A. lyrata* became available recently [62] but too late for inclusion here.) All the RNA-seq data came from a study of RNA editing across *Arabidopsis* species [63] in which total leaf RNA samples were subjected to rRNA depletion and 2 × 100 Illumina sequencing. The *A. lyrata* and *A. halleri* RNA-seq datasets have DDJB accessions DRA007657 and DRA007658 and SRA accessions DRR161380 and DRR161381, respectively.
2. *Brassica***.** We used the *B. rapa* reference with accession GCA_000309985.1 [64, 65] and the *B. oleracea* reference with accession GCA_000695525.1 [66]. The *B. rapa* RNA-seq came from a study of heterosis in Chinese cabbage hybrids, Project PRJNA876066 [67]. The RNA-seq used here was from one of the inbred, non-hybrid parents, C-1 SRR21735970. The *B. oleracea* RNA-seq derived from a study of a Chinese kale allotetraploid, Project PRJNA885390 [68]. The RNA-seq used here was from the diploid parent, CC SRR21778809. Both RNA-seq datasets reflect 2 × 150 Illumina sequencing.
3. *Mus*. We used the *M. musculus* (mouse) C57BL/6 J (‘B6’) reference with accession GCA_000001635.9 [69] and the DBA/2 J (‘D2’) reference with GCA_001624505.1 [70]. All the RNA-seq was 2 × 100 Illumina from a study of gene expression in the retinas of the two mouse strains [71]. We used RNA-seq with accessions SRR8690244 and SRR8690250.
4. *Equus***.** We used the *E. caballus* (horse) reference with accession GCA_002863925.1 [72] and the *E. asinus* (donkey) reference GCA_016077325.2 [50]. Training used 2 × 151 Illumina RNA-seq runs with accessions SRR23724220 and SRR24443170. Validation used data from different individuals that were parts of the same studies, accessions SRR23724221 and SRR24443174. The evaluation on real hybrid data used RNA-seq from a study of pluripotent stem cells [51]: SRR18906505, SRR18906499, SRR18906511.

Raw reads were end-trimmed to remove adapter, low-quality bases, and N base calls using Trim Galore [73] version 0.6.10 using command line ‘trim_galore –cores 4 –trim-n –paired’ plus read 1 and 2 filenames. The read sets were not subjected to duplicate removal, which can be unhelpful unless universal molecular identifiers are present [74].

The mappers selected were ones compared in [10]. The mappers selected use a range of algorithmic approaches including hash tables of k-mer to position in Salmon [4], the FM-index [75, 76] and Burrows-Wheeler transform [77, 78] in Bowtie2 [6, 7], and a suffix array [79] in STAR [8, 9]. Some other mappers had to be excluded because their output formats did not fully support our alignment feature extraction. This included DART [11], Kallisto [5], GSNAP [80], and bwa-mem [47]. The bwa software was not used because its output lacks the XO and XG tags, both optional for SAM files. When used for comparisons, it ran with flag ‘-a bwtsw’ to index the equine genomes and ‘-a is’ to index the smaller references, and with flag ‘-M’ to request a single maximal alignment.

Using Bowtie2 [6, 7] version 2.4.5, references were indexed with default parameters. Read pairs were aligned with command line ‘bowtie2 –no-unal –no-mixed –no-discordant –sensitive –end-to-end –threads 4’ plus options to specify the target sequence filename, the R1 and R2 filenames, and the output filename. We extracted the primary alignment per read pair using SAM/BAM flags. Although Bowtie2 has an option to report multiple alignments per read, the option was not used because the documentation says these alignments are not necessarily the best. HiSat2 [18, 19] version 2.2.1 was used to align RNA paired reads to genomic DNA using all the same options as Bowtie2.

Using STAR [8, 9] version 2.7.10b, references were indexed with command line ‘STAR –runThreadN 4 –runMode genomeGenerate’, plus options to specify the target directory and read filenames. Read pairs were aligned with command line ‘STAR –runThreadN 4 –outSAMattributes NH AS nM NM MD –outSAMtype BAM Unsorted –readFilesCommand gunzip -c’ plus options to specify the index directory and read 1 and 2 filenames. If any process issued recommendations for larger values, such as for parameters limitGenomeGenerateRAM or genomeSAindexNbases, then the program was re-run with the recommended values. For alignments to transcripts, the options ‘–alignIntronMin 100000 –alignIntronMax 0’ were added to preclude splicing. We extracted the primary alignment per read using SAM/BAM flags. Although STAR has an option to report multiple primary alignments, the option was not used because the documentation says no alignments would be reported if the observed number exceeded the given number.

Using Salmon [4] 1.9.0 in mapper mode (i.e. not in conjunction with an aligner), references were indexed with command line ‘salmon index’ and mapped with command line ‘salmon quant –index < index_dir > –libType A –threads 4 –output salmon_out –writeMappings = Aligned.sam’ plus options to specify the read filenames. Recent versions of Salmon recommend using ‘decoys’ to allow Salmon to identify RNA-seq from isoforms missing from the transcriptome, but decoys were not generated or used here. Salmon outputs were not subjected to machine learning because they lacked alignment fields required by our process.

The SAM/BAM format output files were rendered with samtools view [46] and filtered with the flags -f 2 (reads mapped in proper pair), -F 256 (primary alignment per read pair), and -q 1 (minimum map quality 1). Additional file 2: Table S11 shows the effects of filtering. BAM files were parsed by a custom Python script for feature extraction. The script used only read pairs with an alignment to both references. The script relied on the following fields which are optional in BAM files: AS, XM, XO, XG, NM, MD. The script counted events such as mismatches and indels by parsing the CIGAR and MD strings. The script distinguished between such events by whether their base call quality score was maximal. The script accepted the maximum base call quality score encoding (e.g., ‘F’ or ‘K’) as a parameter, and the parameter value was selected by visual inspection of scores in the BAM files in each read set. The feature extractor ignored soft clipping; a cigar string like ‘1S99M’ with one soft-clipped base was treated as 100 aligned bases. Thus, the script could report more mismatches than given in the ‘NM’ field of the BAM file. Alignment spans and read lengths were not used as features since their distributions could be specific to an RNA-seq library or run.

Traditional machine learning was implemented with scikit-learn [81] version 1.2.2. The RandomForestClassifier class was used for random forest models [48]. Feature ranking used the mean decrease in impurity (MDI) method. The GradientBoostingClassifier class was used as a gradient boosting model [82]. The SVC class was used as a support vector machine [83]. The multi-layer perceptron was built with Keras [84].

Read pairs were aligned in the order they appeared in the FASTQ files, which is essentially random. Models were trained using the first **N** read pairs that aligned to both parent references, with the first 80% used for training and the remaining 20% used for evaluation. Each experiment used one pair of RNA-seq FASTQ files, and **N** was adjusted according to data availability. **N** was two million for *Equus* where reads were most copious; **N** was one million for *Arabidopsis*, *Brassica*, and *Mus*; **N** had to be reduced to 400,000 for the one case where STAR aligned few reads to the *Brassica* genomes for unknown reasons. During training, models saw equal numbers of alignments from each parent, and alignments were interleaved such that even and odd samples came from different parents.

We employ several statistics to measure performance. Let TP, FP, TN, and FN represent true positive, false positive, true negative, and false negative rates. For the case of tied alignment scores, one parent was selected randomly. Accuracy = 100*(TP + TN)/(TP + FP + TN + FN) is the most intuitive statistic but it can be misleading for cases of class imbalance. We measure accuracy on class-balanced sets, but nevertheless, we also report sensitivity = 100*TP/(TP + FN), specificity = 100*TN/(TN + FP), F1 = 200*TP/(2*TP + FP + FN), precision = 100*TP/(TP + FP), and Matthews correlation coefficient or MCC = (TP*TN-FP*FN)/sqrt((TP + FP)*(TP + FN)*(TN + FP)*(TN + FN)). AUPRC is the area under the precision-recall curve, a plot of precision vs recall as the classifier’s score threshold varies from 0 to 1. AUROC is the area under the receiver-operator characteristic, a plot of sensitivity vs 1-specificity as the threshold varies. For all these statistics, a higher value is better. We show the map bias as preference for the positive class, “Pos Pref” = (TP + FP)/(TP + FP + TN + FN), for which 50% means no bias. Only the alignment score comparisons generated ties. The number of ties was reported, but the ties were broken randomly for the purpose of generating comparable statistics.

### Supplementary Information

**Additional file 1**. Tables S1–S6.

**Additional file 2**. Tables S7–S15.

## Declarations

### Availability of data and materials

See ENA (https://www.ebi.ac.uk/ena/) for datasets GCA_900078215, GCA_000004255, GCA_000309985, GCA_000695525, GCA_000001635, GCA_001624505, GCA_002863925, GCA_016077325. See SRA (https://www.ncbi.nlm.nih.gov/sra/) for DRR161380, DRR161381, SRR21735970, SRR21778809, SRR8690244, SRR8690250, SRR23724220, SRR24443170, SRR23724221, SRR24443174, SRR18906505, SRR18906499, and SRR18906511. See https://zenodo.org/records/10183055 for source code and notebooks.
