# Supplementary material for: Machine learning on alignment features for parent-of-origin classification of simulated hybrid RNA-seq
Source: BMC Bioinformatics. 2024 Mar 12;25:109. doi: 10.1186/s12859-024-05728-3 (PMC10929170; doi:10.1186/s12859-024-05728-3)
Supplement: Supplementary file 1 — Additional file 1. Tables S1–S6. [file 12859_2024_5728_MOESM1_ESM.docx]

**Supplemental file 1 – Table of Contents**

Page 2.

**Table S1.** Compare machine learning architectures.

Page 3.

**Table S2.** Compare number of trees within random forest.

Page 4.

**Table S3.** Classification of RNA-seq from two mouse strains using alignments to reference transcriptomes.

**Table S4.** Classification of RNA-seq from two mouse strains using alignments to reference genomes.

Page 5.

**Table S5.** Comparison of top 5 features across 12 random forest models.

Page 6.

**Table S6.** Generalization by the *Equus* RNA random forest model.

| *Arabidopsis*  RNA | **A**  Bo+RF | **B**  Bo+GB | **C**  Bo+SVM | **D**  Bo+MLP |
| --- | --- | --- | --- | --- |
| Accuracy:  Sensitivity:  Specificity:  Precision:  F1-score:  MCC:  AUPRC:  AUROC:  Pos Pref:  Ties:  Notebook: | 96.0%  92.1%  99.9%  99.9%  95.8%  0.923  99.7%  99.7%  46.1%  -  **RF_141** | 95.4%  91.7%  99.2%  99.1%  95.3%  0.911  99.6%  99.5%  46.3%  -  **GB_141** | 94.1%  90.7%  97.5%  97.3%  93.9%  0.885  99.0%  98.9%  46.6%  -  **SVM_141b** | 95.9%  92.1%  99.8%  99.8%  95.8%  0.922  99.7%  99.7%  46.1%  -  **MLP_141** |

**Table S1.** Compare machine learning architectures. The table shows classification performance using parental reference transcripts for two species of genus *Arabidopsis*. For the sake of directional statistics like sensitivity, species *A. lyrata* and *A. halleri* were designated as the negative and positive classes, respectively. **A)** Bowtie2 alignments were analyzed with a random forest model using default parameters. This column is shown in the main paper and repeated here for comparison. **B)** Bowtie2 alignments were analyzed with a gradient boosting model using default parameters. **C)** Bowtie2 alignments were analyzed with a support vector machine using default parameters. SVM, as implemented in scikit-learn, was much slower to train. A run on the full dataset of 1 million aligned read pairs per parent exceeded the 24-hour limit on Google CoLab. The results in this column come from a run with 100,000 alignments per parent. **D)** Bowtie2 alignments were analyzed with a multi-layer perceptron designed by the authors. This model used Keras layers Dense(32), Dense(32), Dropout(0.5), Dense(32), Dropout(0.5), Dense(1). All dense layers used the sigmoid activation. Training used the Adam optimizer and 10 epochs.

| *Arabidopsis*  RNA | **A**  Bo+RF | **B**  Bo+GB | **C**  Bo+SVM |
| --- | --- | --- | --- |
| Num Trees: | 100 | 200 | 500 |
| Accuracy:  Sensitivity:  Specificity:  Precision:  F1-score:  MCC:  AUPRC:  AUROC:  Pos Pref:  Ties:  Notebook: | 96.0%  92.1%  99.9%  99.9%  95.8%  0.923  99.7%  99.7%  46.1%  -  **RF_141** | 96.0%  92.1%  99.9%  99.9%  95.8%  0.923  99.7%  99.7%  46.1%  -  **RF_141a** | 96.0%  92.1%  99.9%  99.9%  95.8%  0.923  99.7%  99.7%  46.1%  -  **RF_141b** |

**Table S2.** Compare number of trees within random forest. The table shows classification performance using parental reference transcripts for two species of genus *Arabidopsis*. For the sake of directional statistics like sensitivity, species *A. lyrata* and *A. halleri* were designated as the negative and positive classes, respectively. **A)** Bowtie2 alignments were analyzed with a random forest model using default parameters, including 100 tres (*e.g.* estimators). This column is shown in the main paper and repeated here for comparison. **B)** The number of trees was increased to 200. **C)** The number of trees was increased to 500.

| *Mus*  RNA | **A**  Bowtie2 | **B**  Bo_AS | **C**  Bo_RF | **D**  STAR | **E**  St_AS | **F**  St_RF | **G**  Salmon |
| --- | --- | --- | --- | --- | --- | --- | --- |
| Accuracy:  Sensitivity:  Specificity:  Precision:  F1-score:  MCC:  AUPRC:  AUROC:  Pos Pref:  Ties:  Notebook: | 55.5%  46.4%  64.6%  56.7%  51.0%  0.111  -  -  40.9%  - | 56.4%  54.3%  58.5%  56.7%  55.5%  0.128  -  -  47.9%  82.9% | 56.6%  83.3%  29.8%  54.3%  65.7%  0.155  68.7%  61.8%  76.7%  -  **RF_149** | 53.6%  46.5%  60.7%  54.2%  50.0%  0.072  -  -  42.9%  - | 56.3%  54.7%  57.9%  56.5%  55.6%  0.126  -  -  48.4%  - | 56.3%  81.4%  31.9%  54.4%  65.2%  0.153  65.0%  62.0%  74.7%  83.8%  **RF_158** | 51.3%  34.6%  68.1%  52.0%  41.6%  0.028  -  -  33.3%  - |

**Table S3.** Classification of RNA-seq from two mouse strains using alignments to reference transcriptomes. For directional statistics, *Mus musculus* B6 and D2 were considered the negative and positive classes, respectively.

| *Mus*  DNA | **A**  HiSat2 | **B**  Hi_AS | **C**  Hi_RF | **D**  STAR | **E**  St_AS | **F**  St_RF |
| --- | --- | --- | --- | --- | --- | --- |
| Accuracy:  Sensitivity:  Specificity:  Precision:  F1-score:  MCC:  AUPRC:  AUROC:  Pos Pref:  Ties:  Notebook: | 52.6%  45.7%  59.6%  53.1%  49.1%  0.053  -  -  43.0%  - | 55.7%  51.5%  59.8%  56.2%  53.7%  0.113  -  -  45.8%  77.2% | 56.1%  32.9%  79.4%  61.5%  42.8%  0.139  65.0%  61.7%  26.7%  -  **RF_150** | 52.5%  44.7%  60.4%  53.0%  48.5%  0.051  -  -  42.1%  - | 56.0%  51.8%  60.2%  56.5%  54.1%  0.120  -  -  45.8% | 56.6%  30.0%  83.2%  64.1%  40.9%  0.156  64.7%  61.7%  23.4%  78.3%  **RF_159** |

**Table S4.** Classification of RNA-seq from two mouse strains using alignments to reference genomes. For directional statistics, *Mus musculus* B6 and D2 were considered the negative and positive classes, respectively.

| Feature | | | | Parent | MAT diff | ED diff | P2 R1 HQ MM | HQ INS diff | P2 R2 HQMM | DELS diff | HQ DEL diff | P1 R1 AS | P2 R2 MM |
| --- | --- | --- | --- | --- | --- | --- | --- | --- | --- | --- | --- | --- | --- |
| Rank Total: | | | | 40 | 40 | 23 | 20 | 16 | 15 | 11 | 10 | 4 | 1 |
| Book | Genus | Aligner | Ref |  |  |  |  |  |  |  |  |  |  |
| 141 | Arab. | Bowtie | RNA | 5 | 6 | 4 | 1 | 6 | 2 | 6 | 6 | 3 | 6 |
| 142 | Arab. | STAR | RNA | 6 | 4 | 3 | 1 | 6 | 2 | 5 | 6 | 6 | 6 |
| 143 | Arab. | STAR | DNA | 4 | 5 | 3 | 1 | 6 | 2 | 6 | 6 | 6 | 6 |
| 144 | Arab. | HiSat | DNA | 4 | 5 | 2 | 1 | 6 | 3 | 6 | 6 | 6 | 6 |
| 145 | Brass. | Bowtie | RNA | 1 | 2 | 3 | 6 | 4 | 6 | 6 | 5 | 6 | 6 |
| 148 | Brass. | STAR | RNA | 2 | 1 | 4 | 6 | 3 | 6 | 5 | 6 | 6 | 6 |
| 151 | Brass. | STAR | DNA | 2 | 1 | 4 | 6 | 3 | 6 | 5 | 6 | 6 | 6 |
| 146 | Brass. | HiSat | DNA | 1 | 3 | 2 | 6 | 4 | 6 | 6 | 5 | 6 | 6 |
| 147 | Equ. | Bowtie | RNA | 2 | 1 | 6 | 6 | 3 | 6 | 5 | 4 | 6 | 6 |
| 157 | Equ. | STAR | RNA | 2 | 1 | 6 | 6 | 6 | 6 | 3 | 4 | 5 | 6 |
| 154 | Equ. | STAR | DNA | 2 | 1 | 6 | 6 | 6 | 6 | 3 | 4 | 6 | 5 |
| 153 | Equ. | HiSat | DNA | 1 | 2 | 6 | 6 | 3 | 6 | 5 | 4 | 6 | 6 |

**Table S5.** Comparison of top 5 features across 12 random forest models. Each row describes a random forest trained on a different dataset. The data source for each row is a Jupyter notebook named RF_123, substituting the three digits in the “Book” column. Ranks 1 through 5 indicate first (most important) through fifth, and rank 6 indicates sixth or lower. The columns are ordered left-to-right by descending “Rank Total”, which was computed as ∑(6-rank) over rows in that column. For example, the total of ranks 1, 5, and 6 would be (6-1)+(6-5)+(6-6) = 5+1+0.

| *Equus*  RNA | **A**  Bt_RF | **B**  Bt_RF |
| --- | --- | --- |
| Data set | Primary | Secondary |
| Accuracy:  Sensitivity:  Specificity:  Precision:  F1-score:  MCC:  AUPRC:  AUROC:  Pos Pref:  Ties:  Notebook: | 81.5%  91.3%  71.8%  76.4%  83.2%  0.643  92.1%  91.5%  59.7%  -  **RF_147** | 82.4%  91.4%  73.5%  77.5%  83.9%  0.659  91.8%  91.3%  58.9%  -  **RF_156** |

**Table S6.** Generalization by the *Equus* RNA random forest model. A random forest was trained and tested using Bowtie2 and the reference transcriptomes of genus *Equus*. **A)** The model was trained and tested on the primary dataset which included the horse and donkey RNA-seq runs used in the rest of the main paper. **B)** Without retraining, the model was tested on the secondary dataset which included RNA-seq from different sequencing runs on a different horse and different donkey. Compared to its performance when trained and tested on the primary data, the model performed similarly when trained on the primary and tested on the secondary dataset.
